# Electrochemotherapy Treatment in a Patient with an Extended Basal Cell Carcinoma of the Face: A Case Report

**DOI:** 10.3390/jpm14090984

**Published:** 2024-09-16

**Authors:** Francesco Russano, Davide Brugnolo, Giovanni Bisetto, Paolo Del Fiore, Marco Rastrelli, Simone Mocellin, Luigi Dall’Olmo

**Affiliations:** 1 Soft-Tissue, Peritoneum and Melanoma Surgical Oncology Unit, Veneto Institute of Oncology (IOV), 35128 Padua, Italy; paolo.delfiore@iov.veneto.it (P.D.F.); marco.rastrelli@unipd.it (M.R.); simone.mocellin@iov.veneto.it (S.M.); luigi.dallolmo@iov.veneto.it (L.D.); 2 Department of Surgery, Oncology and Gastroenterology (DISCOG), University of Padua, 35128 Padua, Italy; davide.brugnolo@iov.veneto.it (D.B.); giovanni.bisetto@aopd.veneto.it (G.B.)

**Keywords:** skin cancer, basal cell carcinoma, BCC, electrochemotherapy, ECT, bleomycin, cisplatin, loco-regional treatment

## Abstract

Background. Basal cell carcinomas (BCCs) are common human malignancies with a rising incidence in recent years. While BCCs have a low mortality rate, they are often associated with significant local skin damage characterized by erythema, skin ulceration, and persistent pigmentation. Surgery, radiotherapy, and systemic chemotherapy have traditionally been the principal treatments for these skin injuries. However, electrochemotherapy has recently been proposed as a novel local treatment with promising results for various skin cancers, including BCC, while avoiding the side effects of conventional therapies. ECT involves a local electrical stimulus that enhances cell membrane permeability, thereby enabling the targeted intracellular accumulation of the chemotherapeutic agent. Case Report: We report a case of a 68-year-old man with an ulcerated BCC, following his progress up to 14 months post-ECT treatment, with positive outcomes. Discussion and Conclusions: We achieved a complete clinical response and noted an improvement in the patient’s quality of life. This technique is fast, repeatable, requires minimal hospitalization, and reduces healthcare costs and adverse effects compared to major surgery. Therefore, it can be considered an alternative or complementary approach to traditional surgery for treating BCC of the head and neck.

## 1. Introduction

Basal cell carcinomas (BCCs) are common skin cancers that typically appear on sun-exposed areas of the skin. BCCs are more common in individuals with Fitzpatrick skin types I and II, with an estimated lifetime risk of 30%. The risk of BCC is also associated with light eye color, freckles, and blonde or red hair [1]. UV radiation exposure is the most significant environmental risk factor. Other risk factors include childhood sunburns, a family history of skin cancer, tanning bed use, chronic immunosuppression, photosensitizing drugs, ionizing radiation, and exposure to carcinogenic chemicals, particularly arsenic. Childhood and intense, intermittent sun exposure strongly correlate with BCC development. The estimated incidence of BCC is double in HIV-positive patients and 5 to 10 times higher in organ transplant patients. Approximately half of organ transplant recipients develop a BCC within 10 years post-transplant, with tumors more likely to be the thinner, superficial histologic subtype and occurring in younger patients [2].

While typical cases are diagnosed based on clinical findings, the clinicopathological manifestations can vary. Consequently, a skin biopsy is essential to confirm the diagnosis and assess the risk of recurrence. In treating primary lesions, the initial goal is to ensure complete tumor removal. This can be achieved through various methods, including conventional surgical excision, Mohs micrographic surgery, cryosurgery, electrodesiccation and curettage, topical application of imiquimod or fluorouracil, photodynamic therapy, or radiation therapy [3]. Of these treatments, surgical excision and Mohs surgery are the most commonly used due to their low recurrence rates and the ability to confirm the presence of residual tumor pathologically. However, other treatment options may be preferred based on the patient’s condition, tumor location, and risk of recurrence [4]. Generally, BCCs are slow-growing and rarely metastasize. However, they are locally invasive and can be destructive [2]. In the treatment of metastatic or locally advanced lesions, smoothened inhibitors, which block the activation of the Hedgehog signaling pathway, were recently approved and have shown impressive tumor shrinkage effects. Although the exact prognosis of metastatic BCC has not been thoroughly analyzed, it is likely poor due to the rarity of such cases. However, emerging molecular-targeting agents hold significant therapeutic promise.

Occasionally, there may be locally advanced BCC in aesthetically and functionally sensitive areas, especially in elderly people (e.g., the face). Consequently, surgical treatment with radical intent is not always feasible, and the patient is not always a candidate for medical therapy because of his or her general condition. In such cases, locoregional treatment such as ECT might be considered [5].

Electrochemotherapy (ECT) emerged in the late 1990s in the United States and Europe as a highly effective, minimally invasive locoregional chemotherapy for skin-directed treatment [6]. Recent studies indicate that ECT is linked to clearance rates ranging from 85% to 96% in BCC cases [7]. However, the generalizability of these results is obscured by global variations in study designs, the characteristics of treated patients, and the reporting methods of results.

ECT serves as an alternative treatment for BCC cases deemed unsuitable for standard therapies or experiencing recurrences after standard treatments. This approach involves either intravenous (IV) or intratumoral (IT) injection of a chemotherapeutic agent, such as cisplatin or bleomycin, coupled with locally applied electric pulses. These pulses serve to permeabilize tumor cell membranes, enhancing the cytotoxicity of the chemotherapy agent [8].

ECT has been demonstrated to be safe and effective for treating various types of solid skin tumors. Its application is standardized for skin and subcutaneous localizations, regardless of the tumor’s histological origin [9]. Bleomycin can be administered either intratumorally or systemically, while cisplatin is typically injected intratumorally to optimize outcomes [10]. When the drug is delivered systemically, the electric pulses should be applied to the tumor site during the pharmacokinetic peak, reported to be between 8 and 28 min in humans. For intratumoral applications, the pulses should be delivered from 1 to 10 min after the drug injection [11]. ECT is typically used as a local antitumor therapy for skin or subcutaneous tumors, combining a non-permeant or poorly permeant cytotoxic chemotherapeutic agent (e.g., bleomycin or cisplatin) with short, high-voltage electrical pulses to enhance drug delivery into the cells [12]. Electroporation transiently permeabilizes tumor cell membranes, allowing the diffusion of chemotherapeutic drugs, such as bleomycin or cisplatin, into the cells, thereby increasing the drugs’ cytotoxicity [13]. The cytotoxicity of bleomycin is primarily due to direct DNA damage. The cells are killed because some DNA breaks remain unrepaired, and the cytotoxicity becomes apparent when the cells attempt to divide, as their chromosomes are fragmented. In contrast, quiescent non-dividing cells remain alive [14].

In addition, ECT induces transient local ischemia and permanent vascular damage, reducing tumor vascularization. Besides membrane electroporation, the application of electric pulses to tissues causes a temporary but reversible reduction in blood flow [15].

ECT appears to elicit an autoimmune response that selectively targets neoplastic cells due to the massive shedding of tumor antigens. This induces systemic immunity, which can be further enhanced by additional treatment with biological response modifiers [16].

The most common side effects of ECT include local erythema, skin ulceration, and persistent pigmentation. Here, we describe the clinical course of a patient with locally advanced, ulcerated BCC located on the nose arch and the medial canthus of the right eye.

We applied ECT according to a standardized protocol ESOPE and evaluated the local response in accordance with RECIST criteria (Table 1) after 20, 45, 90, 180 days, and 14 months of follow-up [17].

## 2. Case Report

A 68-year-old man diagnosed with Diffuse Large B-Cell Lymphoma presented to our clinic with rapidly developing, locally advanced lesions on the bridge of his nose and the medial canthus of his right eye. He underwent an incisional biopsy of the lesion, which histologically revealed basal cell carcinoma with desmoplastic features, ulceration, and infiltration into the reticular dermis, along with a slight intratumoral and peritumoral lympho–monocyte reaction.

The patient was presented with several therapeutic options, each with distinct risks and benefits. Radical surgery was suggested as a potentially curative approach but was declined by the patient due to its invasive nature. Similarly, immunotherapy was rejected because of its systemic impact, leading the patient to request a loco-regional approach. While radiotherapy was considered, the significant risk of ocular damage made it less viable due to the lesion’s proximity to the eye. Laser treatments were also evaluated, but their limited effectiveness in achieving sufficient disease control made them a less favorable choice. Consequently, we concluded that ECT was the most suitable approach, with the understanding that if it did not produce the desired outcomes, the patient would consider immunotherapy.

**Figure 1:**
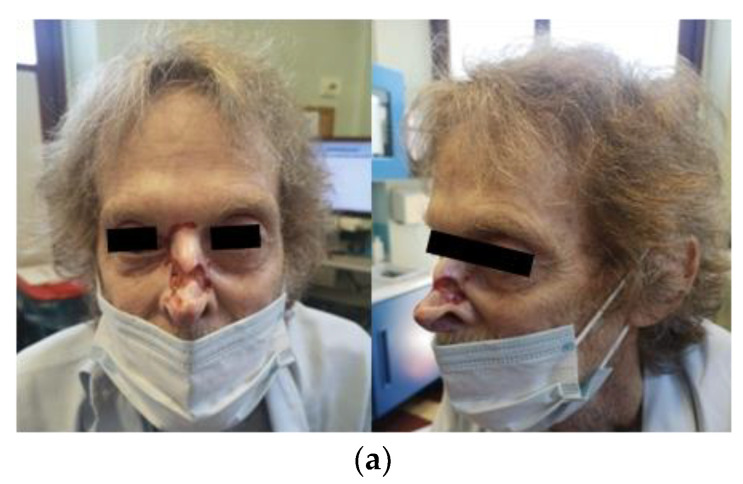

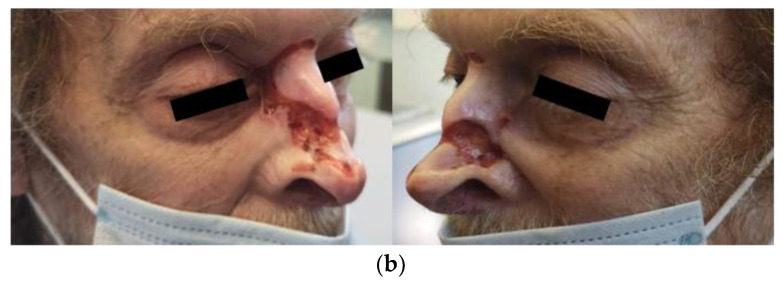
(**a**) Patient before ECT treatment. (**b**) Patient before ECT treatment: magnified view.

On 8 September 2022, we performed ECT with intravenous bleomycin, followed by electroporation using a finger electrode (400 V, 25 pulses) on the ulcerated lesions of the nose, glabellar region, and right eye medial canthus. The patient was discharged the day after the treatment without any complications or pain (Figure 1a,b).

**Figure 2:**
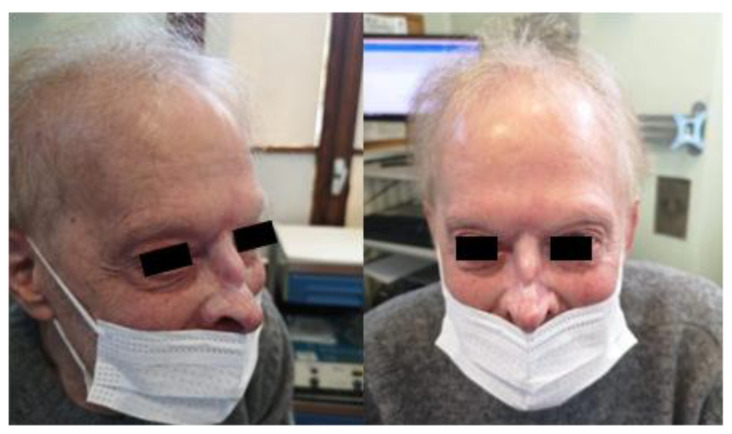
Patient 90 days after ECT treatment.

**Figure 3:**
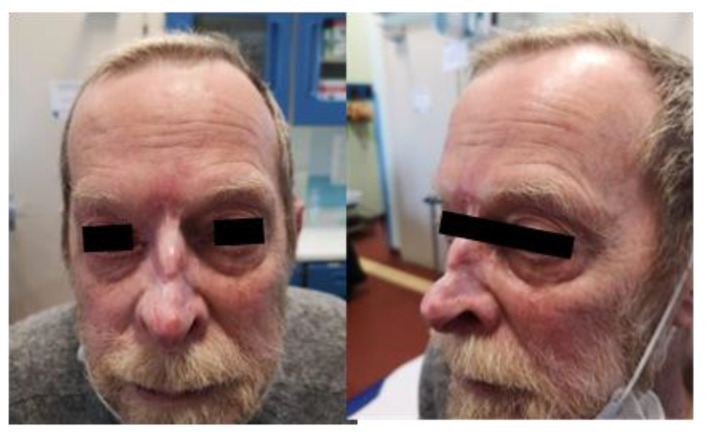
Patient 180 days after ECT treatment.

**Figure 4:**
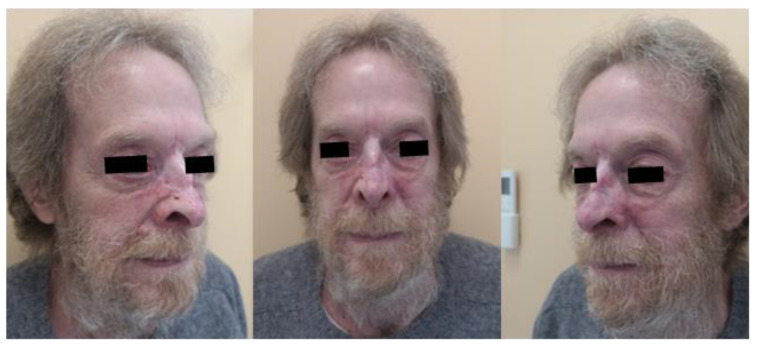
Patient 14 months after ECT treatment.

At a follow-up examination 20 days later, the patient exhibited a mild erythematous rash and healing wounds, with no signs of pain. Forty-five days post-treatment, a second examination showed re-epithelialization of the lesions and resolution of the erythematous rash, indicating a positive response according to the modified RECIST criteria. The patient was monitored at 90 days (Figure 2), 180 days (Figure 3), and 14 months (Figure 4), showing a sustained and complete re-epithelialization of the lesions, allowing clinicians to confirm a complete clinical response. Since no residual disease was clinically visible, it was not necessary to perform a biopsy to evaluate the histological response.

As shown in Table 2 and Table 3, the patient completed a questionnaire related to the Body Image Scale [18] before and after the ECT treatment.

We evaluated the patient’s subjective experience, considering his own perception and its impact on his social life. The patient reported several disease-related aesthetic disturbances and stated that he felt moderately embarrassed and less attractive due to his condition leading him to avoid social situations. After the treatment, however, there was an improvement in his self-perception, and most of complaints completely disappeared. Regarding the appearance of the scars, the patient felt moderately dissatisfied before the treatment, while after the treatment he felt only slightly dissatisfied.

## 3. Discussion

ECT appears to be a reliable and safe approach for controlling BCC skin lesions in patients with ulcerated disease located on the face, yielding good aesthetic results and enhancing the quality of life. This approach is now approved for cutaneous or subcutaneous solid localization of any histological origin and, according to the AIOM guidelines, it can be used in patients with BCC [19].

The established indications for ECT include superficial metastatic melanoma, breast cancer, head and neck skin tumors, nonmelanoma skin cancers, and Kaposi sarcoma. In well-selected patients with oropharyngeal cancers, it can also be used for effective symptom control. Clinical studies have demonstrated that ECT is an efficient and safe method for local disease control in patients with BCC and other cutaneous tumors; in particular an overall response of 96% and complete response rate of 85% have been achieved for BCC [7,20,21,22]. The main objective of ECT treatment is to make cancer cells permeable to chemotherapy when the drug is present at the tumor site. This allows cytotoxic drugs that are normally non-permeable or have poor permeability to enter the cells and kill them at systemic concentrations lower than those typically required, thereby reducing the risk of treatment-related side effects. Bleomycin can be delivered either intratumorally or systemically, while cisplatin should be injected intratumorally. When performing a systemic injection, the electric pulses should be delivered during the pharmacokinetic peak, which typically occurs between 8 and 28 min [23]. For intratumoral injection, the pulses should be delivered within 1 to 10 min, with the optimal response typically observed within 5 min [11].

Bleomycin and Cisplatin are both hydrophilic drugs, meaning that under normal conditions, they cannot penetrate the phospholipid bilayer of human cells. Electroporation transiently permeabilizes the membranes of tumor cells, thereby facilitating the diffusion of these two chemotherapeutic drugs into the cytoplasm. The toxicity of bleomycin and cisplatin primarily arises from direct DNA damage. Additionally, ECT induces transient local ischemia and vascular damage in the tumor bed, which further enhances the cytotoxic effect on the target tissue [15]. ECT induces a reduction in blood flow at the tumor site, effectively trapping the drug in that area, which can enhance the treatment’s effectiveness. Additionally, ECT appears to stimulate an immune response that may contribute to its efficacy in local control, although this effect has yet to be conclusively demonstrated [16].

The most common side effects include local erythema, skin ulceration, and persistent pigmentation. Typically, the treatment is painless [24]. The main limitations of the procedure include its inability to be applied for deep tumors, known allergy to bleomycin, and the fact that it is not indicated for pregnant women or patients with severe pulmonary fibrosis. Other contraindications, such as the presence of a pacemaker or anticoagulant therapy [6], are no longer exclusion criteria with proper safety systems in place.

In this case report, we illustrate the efficacy and safety of ECT treatment in a patient with extensive basal cell carcinoma of the face. The patient achieved a complete clinical response to the disease without undergoing major reconstructive surgeries. After the ECT treatment, all lesions disappeared and complete re-epithelialization occurred; therefore, performing biopsies was not necessary, as there was no visible residual disease.

In addition to achieving a complete clinical response, the patient also expressed satisfaction with the treatment from a subjective and aesthetic standpoint [25], as evidenced by the patient’s responses on the Body Image Scale. Between the two assessments, all parameters either improved or remained unchanged. After the treatment, the patient reported feeling less embarrassed and more physically attractive. Furthermore, there was an improvement in relationships with other people, and the patient expressed a high degree of satisfaction with the appearance of his scar.

The quality of life improved, and a complete response was achieved, which was sustained for at least 14 months. This method demonstrated effectiveness, safety, speed, and relative affordability. It can be regarded as a more conservative treatment option, with documented enhancements in aesthetics and quality of life.

## 4. Conclusions

ECT with bleomycin (or cisplatin) is an effective and safe therapy for localized tumor control of primary and recurrent tumors of the skin (melanoma and nonmelanoma skin cancer), as well as cutaneous and subcutaneous tumor nodules of various histologies. The treatment is repeatable and does not preclude the subsequent use of traditional therapies. This treatment may be considered for locally aggressive tumors of the face or other exposed areas of the body, as demonstrated in this case report. We achieved a complete clinical response and also noted an improvement in the patient’s reported quality of life from a subjective standpoint.

The main advantages of this technique include its speed, repeatability, minimal hospitalization times, resulting in lower costs for the healthcare system, and a reduced incidence of potential adverse effects associated with major surgery.

For these reasons, this technique can be viewed as an alternative or complementary approach to traditional surgery for the treatment of BCC of the head and neck.

## Figures and Tables

**Table 1:** Response evaluation criteria in solid tumors (RECISTs).

Response	CT—Mass Size
Complete	Lesion disappearance (scar) or <25% original size
Partial	>30% decrease in the sum LD of target lesions
Stable lesion	<30% decrease in the sum LD of target lesions
Progression	Increase of >20% in sum LD of target lesions

**Table 2:** Body Image Scale before ECT treatment [18].

Body Image Scale (Before Treatment)		Not at All	A Little	Moderately	Very Much	Don’t Know
1	Did you ever feel embarrassed about your physical appearance?			X		
2	Have you felt less physically attractive as a result of your disease or treatment?			X		
3	Have you been dissatisfied with your appearance when dressed?			X		
4	Have you been feeling less masculine as a result of your disease or treatment?	X				
5	Did you find it difficult to look at yourself naked?	X				
6	Have you been feeling less sexually attractive as a result of your disease or treatment?			X		
7	Do you avoid meeting people because of the way you feel about your appearance?			X		
8	Do you feel the disease or treatment has left your body less whole?	X				
9	Have you felt dissatisfied with your body?	X				
10	Have you felt dissatisfied with the appearance of your scar?			X		

**Table 3:** Body Image Scale after ECT treatment [18].

Body Image Scale (After Treatment)		Not at All	A Little	Moderately	Very Much	Don’t Know
1	Did you ever feel embarrassed about your physical appearance?		X			
2	Have you felt less physically attractive as a result of your disease or treatment?		X			
3	Have you been dissatisfied with your appearance when dressed?		X			
4	Have you been feeling less masculine as a result of your disease or treatment?	X				
5	Did you find it difficult to look at yourself naked?	X				
6	Have you been feeling less sexually attractive as a result of your disease or treatment?	X				
7	Do you avoid meeting people because of the way you feel about your appearance?	X				
8	Do you feel the disease or treatment has left your body less whole?	X				
9	Have you felt dissatisfied with your body?	X				
10	Have you felt dissatisfied with the appearance of your scar?		X			

## Data Availability

The original contributions presented in the study are included in the article, further inquiries can be directed to the corresponding author.
